# Development and validation of lymph node ratio-based nomograms for primary duodenal adenocarcinoma after surgery

**DOI:** 10.3389/fonc.2022.962381

**Published:** 2022-10-04

**Authors:** Jingxiang Shi, Sifan Liu, Jisen Cao, Shigang Shan, Jinjuan Zhang, Yijun Wang

**Affiliations:** ^1^ Department of Hepatobiliary Surgery, The Third Central Hospital of Tianjin, Tianjin, China; ^2^ Tianjin Key Laboratory of Extracorporeal Life Support for Critical Diseases, The Third Central Hospital of Tianjin, Tianjin, China; ^3^ Artificial Cell Engineering Technology Research Center, The Third Central Hospital of Tianjin, Tianjin, China; ^4^ Tianjin Institute of Hepatobiliary Disease, The Third Central Hospital of Tianjin, Tianjin, China; ^5^ School of Statistics, Tianjin University of Finance and Economics, Tianjin, China

**Keywords:** lymph node ratio, nomogram, primary duodenal adenocarcinoma, overall survival, cancer-specific survival

## Abstract

**Background:**

The prediction models for primary duodenal adenocarcinoma (PDA) are deficient. This study aimed to determine the predictive value of the lymph node ratio (LNR) in PDA patients and to establish and validate nomograms for predicting overall survival (OS) and cancer-specific survival (CSS) for PDAs after surgical resection.

**Methods:**

We extracted the demographics and clinicopathological information of PDA patients between 2004 and 2018 from the Surveillance, Epidemiology and End Results database. After screening cases, we randomly divided the enrolled patients into training and validation groups. X-tile software was used to obtain the best cut-off value for the LNR. Univariate and multivariate Cox analyses were used in the training group to screen out significant variables to develop nomograms. The predictive accuracy of the nomograms was evaluated by the concordance index (C-index), calibration curves, area under the receiver operating characteristic curve (AUC) and decision curve analysis (DCA). Finally, four risk groups were created based on quartiles of the model scores.

**Results:**

A total of 978 patients were included in this study. The best cut-off value for the LNR was 0.47. LNR was a negative predictive factor for both OS and CSS. Age, sex, grade, chemotherapy and LNR were used to construct the OS nomogram, while age, grade, chemotherapy, the number of lymph nodes removed and LNR were incorporated into the CSS nomogram. The C-index, calibration curves and AUC of the training and validation sets revealed their good predictability. DCA showed that the predictive value of the nomograms was superior to that of the American Joint Committee on Cancer (AJCC) TNM staging system (8th edition). In addition, risk stratification demonstrated that patients with higher risk correlated with poor survival.

**Conclusions:**

The LNR was an adverse prognostic determinant for PDAs. The nomograms provided an accurate and applicable tool to evaluate the prognosis of PDA patients after surgery.

## Introduction

Primary duodenal adenocarcinoma (PDA), which arises from duodenal mucosal glandular epithelial cells, is a rare malignant tumour (1). According to epidemiological investigation, its incidence is no more than 0.5 in 100,000, accounting for only 0.5% of all gastrointestinal tumours (2, 3). The majority of PDAs occur in the second portion of the duodenum, followed by the first portion (4). Due to the adjacency of anatomical position and the same surgical approach, cancers from the head of pancreas, the distal common bile duct, the ampulla of Vater and the descending portion of the duodenum called “periampullary carcinoma” have often been studied together (5). However, the biological features and prognosis of these tumours are different. Therefore, it is necessary to study PDA as a unique entity.

Surgical resection is the main curative treatment for PDAs (6). Pancreaticoduodenectomy or segmental resection is performed in most cases (7). One meta-analysis demonstrated that surgery might greatly improve survival compared with nonsurgery (5-year overall survival rate, 43.4% vs 2.5%) (8). Poultsides et al. (9) pointed out that the 5-year overall survival (OS) rate after resection of PDA was 48%. Another study indicated that the 5-year cancer-specific survival (CSS) rate after surgery was 43% (3). However, due to the low incidence of this disease and the small sample size of clinical research, there are few studies on prediction models for PDA patients after surgery.

The lymph node ratio (LNR), which refers to the proportion of positive lymph nodes in the total examined nodes, has shown its importance for the prognostic prediction of several malignancies in recent years. Zhou et al. (10) pointed out that the LNR was an important prognostic factor for non-small-cell lung cancer. Macedo et al. (11) indicated that the LNR was a better prognostic tool than the TNM system in colorectal cancer. Our previous study demonstrated that it was an important predictor of poor survival in pancreatic neuroendocrine tumours (12). However, there are no published reports about nomograms for predicting the survival of PDA patients combined with LNR data.

In this study, based on data from the Surveillance, Epidemiology, and End Results (SEER) database, we identified the effect of the LNR on the prognosis of patients with PDA. We further established and validated nomograms to predict 1-, 3-, and 5-year OS and CSS rates for PDAs after surgery.

## Materials and methods

### Data collection

The SEER database is a population-based database supported by the National Cancer Institute in the USA and contains a large amount of evidence-based medical data (13). We used SEER*Stat software (version 8.3.9.2) to extract data from the SEER database. The Incidence SEER Research Plus Data, 18 Registries, Nov 2020 Sub (2000–2018) dataset was selected for analysis (username for log in: 15881-Nov2020). Patients diagnosed with PDA between 2004 and 2018 were confirmed retrospectively. The selection parameters in the software were as follows: Site and Morphology, “Primary Site – labelled” (C17.0-Duodenum) and “ICD-O-3 Hist/behav” (8140/3, 8143/3, 8144/3, 8210/3, 8211/3, 8220/3, 8221/3, 8255/3, 8260/3-8263/3, 8310/3, 8323/3, 8480/3, 8481/3, 8574/3, 8576/3). The extracted demographic variables included age, sex, patient ID, year of diagnosis, race and marital status. The corresponding clinicopathological variables were as follows: histologic type, grade, diagnostic confirmation, American Joint Committee on Cancer (AJCC) TNM staging system, T stage, N stage, M stage, tumour size, surgery at the primary site, scope of regional lymph node surgery, radiation recode, chemotherapy recode, regional nodes examined, positive regional nodes, survival months, SEER cause-specific death classification, vital status recode, first malignant primary indicator and sequence number.

### Data processing

The exclusion criteria were as follows: 1) patients who did not undergo surgery; 2) patients who did not have regional lymph nodes removed during surgery; 3) patients who had multiple primary tumours; and 4) missing or unknown clinical information.

We treated age as a continuous variable and other factors as categorical variables. Patients who were single, separated, divorced or widowed were regarded as unmarried. Tumour grade was defined as Grade I (well differentiated), Grade II (moderately differentiated), Grade III (poorly differentiated) and Grade IV (undifferentiated or anaplastic) according to the SEER tumour grade system. The TNM staging system was adjusted based on the eighth edition of AJCC. OS was defined as the time from the date of diagnosis to the date of death by any cause or last follow-up, and CSS was defined as the time from the date of diagnosis to the date of death caused by PDA (12).

### Construction and validation of the nomograms

The enrolled patients were randomly assigned to a training set and a validation set at a ratio of 7:3. For the training cohort, the “survival” package in R software was used for univariate and multivariate Cox regression analysis to screen significant variables (14). After that, the “rms” package was used to construct nomograms based on the results of multivariate analysis (14). The validation cohort was treated as an external validation of the nomograms.

We calculated the concordance index (C-index) and the area under the receiver operating characteristic curve (AUC) of the training and validation groups to evaluate the discrimination ability of the nomograms (15). Calibration curves were plotted to assess the calibration ability (16). In addition, we used decision curve analysis (DCA) to evaluate the clinical usefulness of the nomograms (17). Furthermore, DCA was performed to compare the AJCC staging system (8th edition), LNR and the nomograms.

### Statistical analysis

Statistical analyses were performed as described in our previous study (12). Continuous variables were reported as the mean with standard deviation. Categorical variables were presented as frequencies and percentages. X-tile software was used to determine the optimal cut-off value of the LNR (18). Survival curves (for both OS and CSS) were generated using the Kaplan–Meier method, and the differences in groups were analysed using the log-rank test. Variables with *P* < 0.2 in univariate analysis were considered for generating multivariate analysis. Factors with *P* < 0.05 in multivariate analysis were entered into the nomograms. Corresponding 95% confidence intervals (CIs) and hazard ratios (HRs) were calculated. We used Pearson’s correlation to detect collinearity among the variables. A correlation coefficient < 0.7 between two variables was regarded as no multicollinearity (19). *P* < 0.05 was defined as statistically significant. All statistical tests were performed using R statistical software (version 4.1.2, https://www.r-project.org, Vienna, Austria).

## Results

### Patient characteristics

**Figure 1:**
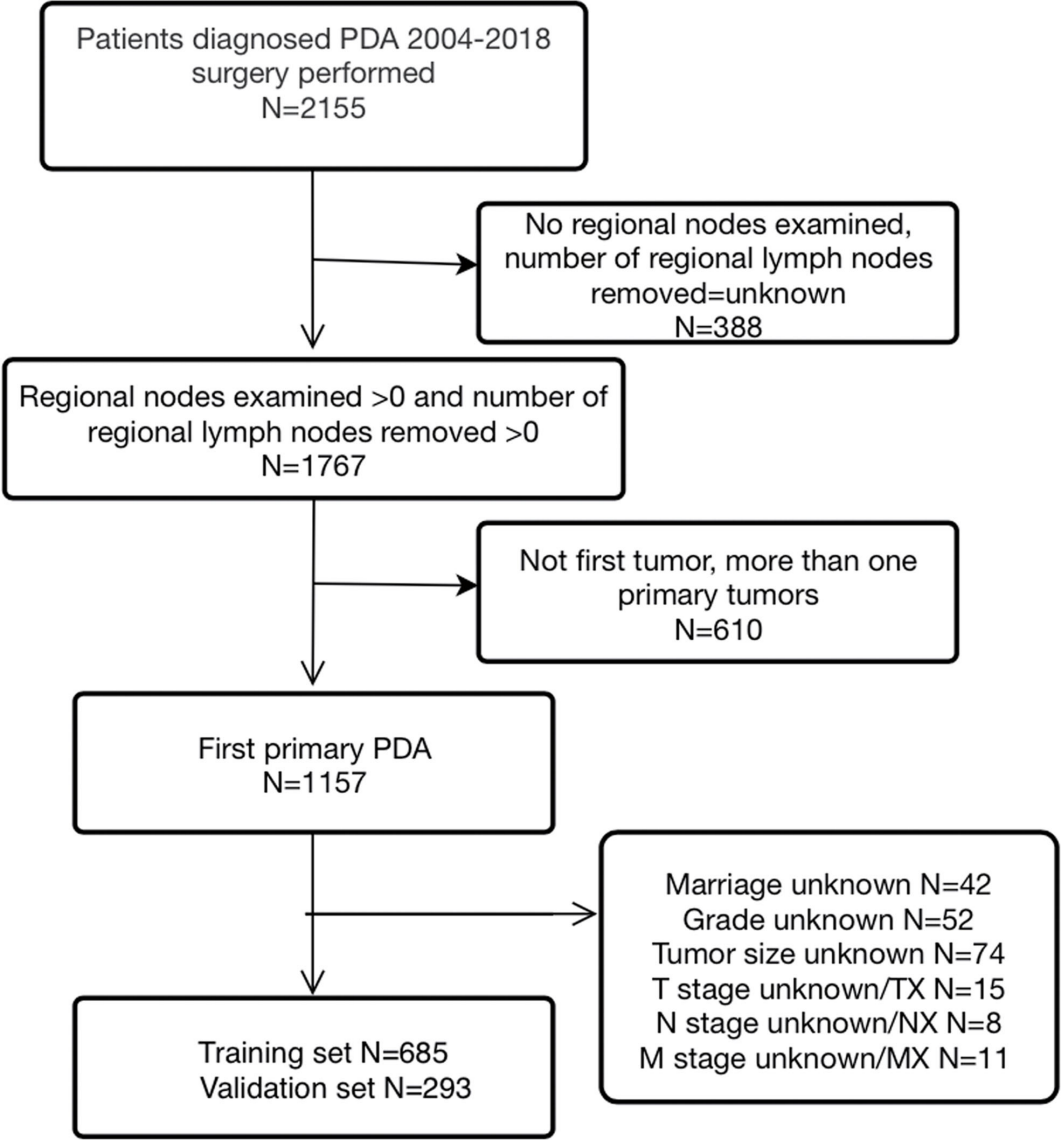
The flow chart of included patients.

**Figure 2:**
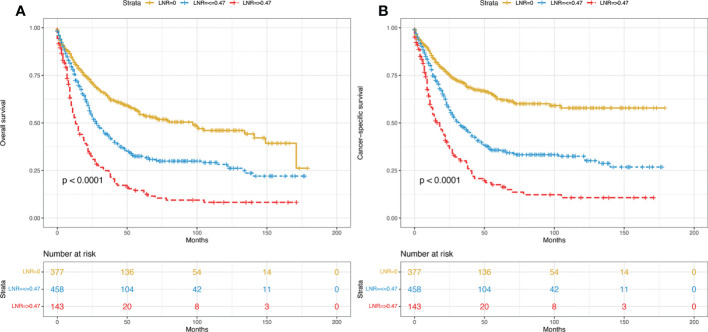
Kaplan–Meier curves of OS **(A)** and CSS **(B)** for all included patients stratified by LNR. OS, overall survival; CSS, cancer-specific survival; LNR, lymph node ratio.

From 2004 to 2018, a total of 978 eligible patients from the SEER database were included in this study. Among them, 685 cases were randomly allocated to the training group, while 293 cases were allocated to the validation group. The study procedure is shown in **Figure 1**. The demographic and clinicopathological characteristics of the enrolled patients are summarized in **Table 1**. For the overall group, the median age was 65 years, and the median follow-up time was 38 months (range, 1-179 months). By the end of the last follow-up, 326 patients (47.6%) had died from PDA, and 56 patients (8.2%) had died from other causes in the training set. For the validation set, the numbers were 138 (47.1%) and 26 (8.9%), respectively. The 1-, 3-, and 5-year OS rates for the training group and the validation group were 75.8%, 55.3%, 47.9% and 76.8%, 53.6%, 47.4%. The corresponding 1-, 3-, and 5-year CSS rates for the two groups were 79.1%, 61.0%, 54.5% and 80.9%, 60.8%, 55.3%, respectively. There were no significant differences between the two groups. The best cut-off value for the LNR was 0.47. Thus, we divided the total patients into three groups (LNR1: 0, LNR2: ≤ 0.47 and LNR3: > 0.47). Patients with a lower LNR were associated with better OS and CSS (**Figures 2A, B** **)**.

**Table 1:** Patient demographics and clinicopathological characteristics.

Variables	Training group	Validation group
Age (year)	64.72 ± 12.75	65.85 ± 12.00
Sex		
Female	315 (46.0)	144 (49.1)
Male	370 (54.0)	149 (50.9)
Race		
White	529 (77.2)	215 (73.4)
Black	96 (14.0)	54 (18.4)
Others	60 (8.8)	24 (8.2)
Marriage		
Married	445 (65.0)	185 (63.1)
Unmarried	240 (35.0)	108 (36.9)
Grade		
I	57 (8.3)	21 (7.2)
II	355 (51.8)	165 (56.3)
III	263 (38.4)	104 (35.5)
IV	10 (1.5)	3 (1.0)
Tumour size (cm)		
≤2	94 (13.7)	32 (10.9)
2-4	293 (42.8)	125 (42.7)
>4	298 (43.5)	136 (46.4)
T stage		
T1	36 (5.3)	18 (6.1)
T2	42 (6.1)	27 (9.2)
T3	257 (37.5)	109 (37.2)
T4	350 (51.1)	139 (47.4)
N stage		
N0	245 (35.8)	128 (43.7)
N1	318 (46.4)	127 (43.3)
N2	122 (17.8)	38 (13.0)
M stage		
M0	630 (92.0)	273 (93.2)
M1	55 (8.0)	20 (6.8)
Radiotherapy		
Yes	89 (13.0)	45 (15.4)
No	596 (87.0)	248 (84.6)
Chemotherapy		
Yes	348 (50.8)	156 (53.2)
No	337 (49.2)	137 (46.8)
Lymph nodes removed		
<15	367 (53.6)	145 (49.5)
≥15	318 (46.4)	148 (50.5)
AJCC TNM stage		
I	54 (7.9)	30 (10.2)
IIA	101 (14.7)	55 (18.8)
IIB	87 (12.7)	39 (13.3)
IIIA	284 (41.5)	116 (39.6)
IIIB	104 (15.2)	33 (11.3)
IV	55 (8.0)	20 (6.8)
LNR		
0	262 (38.2)	115 (39.2)
≤0.47	324 (47.3)	134 (45.7)
>0.47	99 (14.5)	44 (15.0)

LNR, lymph node ratio.

### Nomogram construction

**Figure 3:**
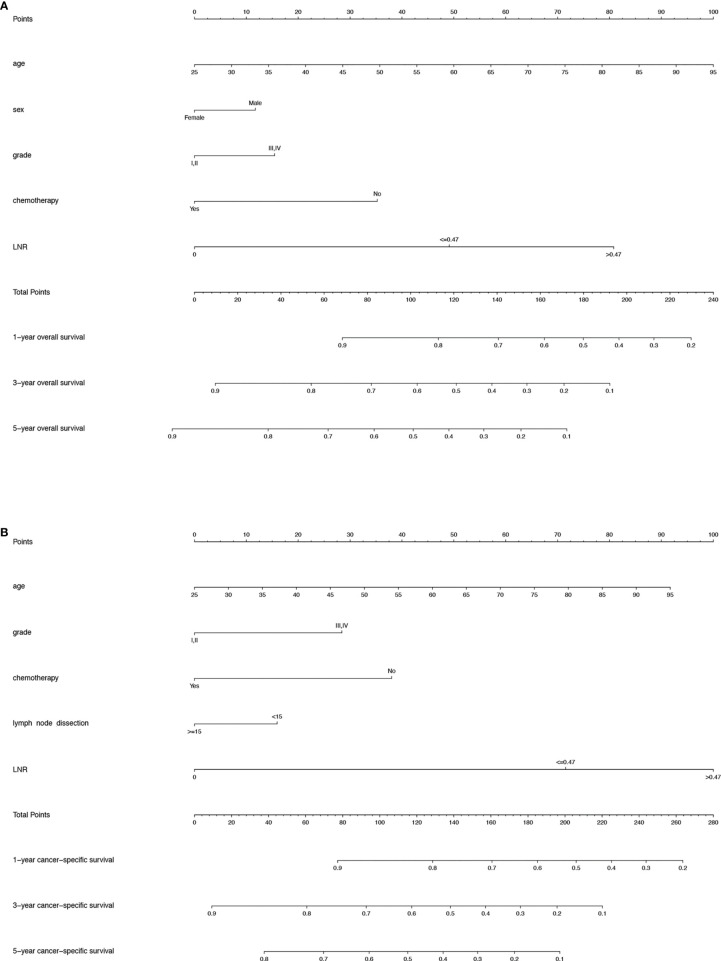
**(A)** Nomogram predicting the 1-, 3- and 5-year OS rates for PDA patients after surgery. **(B)** Nomogram predicting the 1-, 3- and 5-year CSS rates for PDA patients after surgery. OS, overall survival; PDA, primary duodenal adenocarcinoma; CSS, cancer-specific survival.

For the training cohort, the following variables were included in the univariate analysis: age, sex, race, marital status, grade, tumour size, T stage, N stage, M stage, radiotherapy, chemotherapy, the number of lymph nodes removed and LNR. The factors with significant differences were incorporated into the multivariate Cox regression analysis. The multivariate analyses identified that age, sex, grade, chemotherapy and LNR were significantly associated with OS and that age, grade, chemotherapy, the number of lymph nodes removed and LNR were significantly associated with CSS (**Tables 2**, **3**). Therefore, nomograms for predicting the 1-, 3-, and 5-year OS and CSS rates were established based on these variables (**Figures 3A, B**). Each variable was given a score on the points scale. The survival probability of a patient can be easily calculated by adding the scores of each selected factor. There was no collinearity among the screened variables for the overall dataset, the training or the validation sets (**Supplementary Figure S1**).

**Table 2:** Univariate and multivariate analysis of overall survival (OS) in the training set.

	Univariate analysis		Multivariate analysis	
	HR (95% CI)	*P*	HR (95% CI)	*P*
Age (year)	1.03 (1.02-1.04)	<0.001	1.02 (1.01-1.03)	<0.001
Sex				
Female	0.74 (0.60-0.90)	0.003	0.79 (0.64-0.97)	0.022
Male	Reference		Reference	
Race				
White	Reference			
Black	0.95 (0.71-1.29)	0.750		
Others	1.23 (0.86-1.76)	0.252		
Marriage				
Married	Reference			
Unmarried	1.12 (0.91-1.39)	0.275		
Grade				
I, II	Reference		Reference	
III, IV	1.43 (1.17-1.75)	<0.001	1.44 (1.17-1.78)	0.009
Tumour size (cm)				
≤2	Reference			
2-4	1.18 (0.87-1.60)	0.290		
>4	1.05 (0.77-1.43)	0.756		
T stage				
T1	Reference			
T2	0.75 (0.41-1.39)	0.368		
T3	0.91 (0.57-1.45)	0.695		
T4	0.93 (0.59-1.48)	0.766		
N stage				
N0	Reference			
N1	0.97 (0.77-1.21)	0.768		
N2	1.14 (0.86-1.51)	0.369		
M stage				
M0	Reference			
M1	0.95 (0.65-1.38)	0.780		
Radiotherapy				
Yes	0.71 (0.52-0.97)	0.033	1.06 (0.75-1.50)	0.729
No	Reference		Reference	
Chemotherapy				
Yes	0.68 (0.55-0.83)	<0.001	0.49 (0.38-0.62)	<0.001
No	Reference		Reference	
Lymph nodes removed				
<15	Reference		Reference	
≥15	0.73 (0.59-0.90)	0.003	0.82 (0.65-1.02)	0.079
LNR				
0	Reference		Reference	
≤0.47	1.66 (1.31-2.09)	<0.001	2.40 (1.85-3.12)	<0.001
>0.47	3.08 (2.31-4.11)	<0.001	3.67 (2.67-5.03)	<0.001

LNR, lymph node ratio.

**Table 3:** Univariate and multivariate analysis of cancer-specific survival (CSS) in the training set.

	Univariate analysis		Multivariate analysis	
	HR (95% CI)	*P*	HR (95% CI)	*P*
Age (year)	1.03 (1.02-1.03)	<0.001	1.02 (1.01-1.03)	<0.001
Sex				
Female	0.81 (0.65-1.01)	0.062	0.90 (0.72-1.12)	0.336
Male	Reference		Reference	
Race				
White	Reference			
Black	0.95 (0.69-1.31)	0.755		
Others	1.16 (0.78-1.71)	0.460		
Marriage				
Married	Reference			
Unmarried	1.10 (0.87-1.37)	0.437		
Grade				
I, II	Reference		Reference	
III, IV	1.69 (1.36-2.09)	<0.001	1.68 (1.34-2.11)	<0.001
Tumour size (cm)				
≤2	Reference		Reference	
2-4	1.41 (0.99-2.00)	0.050	1.40 (0.99-1.99)	0.059
>4	1.19 (0.84-1.69)	0.332	1.20 (0.84-1.71)	0.310
T stage				
T1	Reference			
T2	1.01 (0.51-1.97)	0.987		
T3	0.96 (0.56-1.64)	0.876		
T4	1.14 (0.67-1.94)	0.621		
N stage				
N0	Reference			
N1	1.06 (0.83-1.35)	0.651		
N2	1.19 (0.87-1.62)	0.280		
M stage				
M0	Reference			
M1	1.00 (0.68-1.49)	0.987		
Radiotherapy				
Yes	0.76 (0.54-1.05)	0.094	1.03 (0.72-1.48)	0.857
No	Reference		Reference	
Chemotherapy				
Yes	0.76 (0.61-0.94)	0.012	0.49 (0.38-0.64)	<0.001
No	Reference		Reference	
Lymph nodes removed				
<15	Reference		Reference	
≥15	0.73 (0.58-0.91)	0.006	0.77 (0.60-0.98)	0.034
LNR				
0	Reference		Reference	
≤0.47	1.98 (1.52-2.57)	<0.001	2.92 (2.18-3.90)	<0.001
>0.47	3.66 (2.67-5.03)	<0.001	4.25 (3.01-6.00)	<0.001

LNR, lymph node ratio.

### Nomogram validation

**Figure 4:**
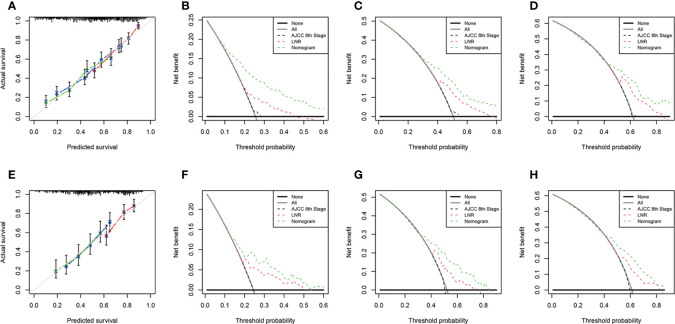
Calibration curves for OS prediction in the training cohort **(A)** and the validation cohort **(E)**. Decision curve analysis of the AJCC 8th edition staging system, nomogram and LNR for the 1- **(B)**, 3- **(C)** and 5-year **(D)** OS rates of PDA patients from the training cohort. Decision curve analysis of the AJCC 8th edition staging system, nomogram and LNR for the 1- **(F)**, 3- **(G)** and 5-year **(H)** OS rates of PDA patients from the validation cohort. OS, overall survival; LNR, lymph node ratio; PDA, primary duodenal adenocarcinoma. For calibration curves, red, blue and green lines represent 1, 3, and 5 years, respectively.

**Figure 5:**
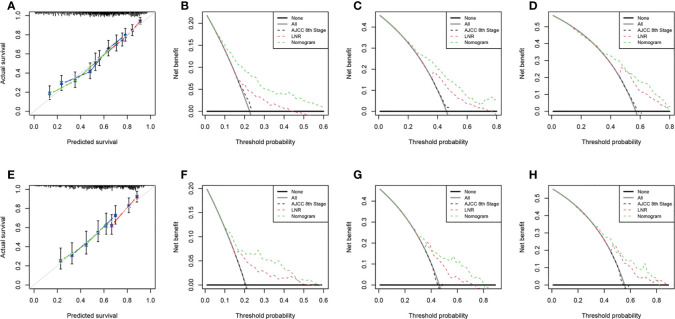
Calibration curves for CSS prediction in the training cohort **(A)** and the validation cohort **(E)**. Decision curve analysis of the AJCC 8th edition staging system, nomogram and LNR for the 1- **(B)**, 3- **(C)** and 5-year **(D)** CSS rates of PDA patients from the training cohort. Decision curve analysis of the AJCC 8th edition staging system, nomogram and LNR for the 1- **(F)**, 3- **(G)** and 5-year **(H)** CSS rates of PDA patients from the validation cohort. CSS, cancer-specific survival; LNR, lymph node ratio; PDA, primary duodenal adenocarcinoma. For calibration curves, red, blue and green lines represent 1, 3, and 5 years, respectively.

We performed internal and external verification of the two nomograms. In the internal validation, the C-index for OS prediction was 0.697 (95% CI, 0.670 to 0.724), while for CSS, it was 0.700 (95% CI, 0.671 to 0.729). In the external validation, the C-index for OS prediction was 0.669 (95% CI, 0.629 to 0.709), while for CSS, it was 0.674 (95% CI, 0.626 to 0.722). The calibration curves of the OS nomogram showed high consistency between the predicted and observed survival probability in both the training (**Figure 4A**) and validation (**Figure 4E**) cohorts. Similarly, the calibration plots of the CSS nomogram were close to the standard curves in the two sets (**Figures 5A, E**). The AUC values for predicting 1-, 3-, and 5-year OS rates were 0.767, 0.717 and 0.704 in the training group (**Supplementary Figure S2A**) and 0.705, 0.722 and 0.715 in the validation group (**Supplementary Figure S2B**). With regard to the 1-, 3-, and 5-year CSS rates, the AUC values were 0.762, 0.733 and 0.703 in the training group (**Supplementary Figure S2C**) and 0.764, 0.715 and 0.706 in the validation group (**Supplementary Figure S2D**). For both the training and validation sets, the DCA curves for OS (**Figures 4B–D, F–H**) and CSS (**Figures 5B-D, F-H**) indicated that our nomograms exhibited optimal net benefit with a wider range of threshold probability compared to the AJCC staging system (8th edition), and LNR had good prediction ability regarding patient prognosis.

### Risk stratification

**Figure 6:**
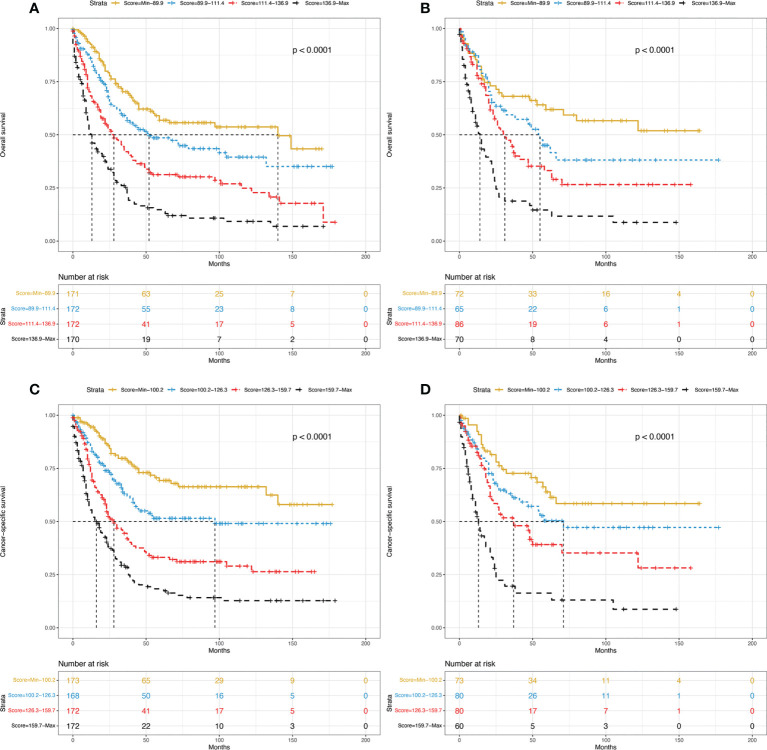
Kaplan–Meier curves of OS for the training group **(A)** and validation group **(B)** stratified by different risk scores. Kaplan–Meier curves of CSS for the training group **(C)** and validation group **(D)** stratified by different risk scores. OS, overall survival; CSS, cancer-specific survival.

Finally, we performed risk stratification for PDA patients based on the two established nomograms. They were categorized into four groups according to the scores for OS (Min-89.9, 89.9-111.4, 111.4-136.9, 136.9-Max) or the scores for CSS (Min-100.2, 100.2-126.3, 126.3-159.7, 159.7-Max). The Kaplan–Meier survival curves showed that statistically significant differences in OS (**Figures 6A, B** **)** and CSS (**Figures 6C, D** **)** were observed among the four different risk groups in both the training and validation sets.

## Discussion

PDA is a rare gastrointestinal malignancy. Patients are usually diagnosed at an advanced stage due to a lack of specific symptoms, which leads to adverse prognoses (3). Considering the low prevalence of this disease and the limited number of clinical studies, there is no consensus on prognostic factors. Therefore, we used a large dataset collected by the SEER program to identify the factors affecting the survival of PDA patients after surgery. Furthermore, we developed nomograms to predict OS and CSS for these patients. Given the significance of LNR, we analysed it separately and integrated it into the nomograms according to the multivariate Cox regression analysis. The relatively high C-indices and values of AUC in the training and validation sets demonstrated that the nomograms displayed good ability in predicting OS and CSS. The calibration curves showed excellent agreement between the predicted and actual survival, which further ensured their accuracy and reliability.

It is well established that lymph node status (yes/no presence of metastatic node) is an important prognostic determinant in PDA patients after operation. Sakamoto et al. (20) suggested that lymph node metastasis was a negative predictive factor for OS. Meijer et al. (21) reported that lymph node involvement correlated with poor survival. However, lymph node status ignores the different prognoses of lymph node-positive cases, which may lead to confusion in such patients. To overcome this limitation, LNR emerged as a substitute for lymph node status. Its effect on survival has been confirmed by many neoplasms, such as gastric, pancreatic, and ampullary cancer (22–24). With respect to PDA, Jensen et al. (25) pointed out that LNR > 0.2 was associated with increased mortality. Tran et al. (26) indicated that the LNR was an important predictor of lower OS. Liang et al. (27) identified that LNR > 0.4 was associated with decreased survival. Another study showed that patients with high LNR levels had shorter OS (9). Our study, in accordance with previous reports, demonstrated that LNR was an adverse independent prognostic factor for both OS and CSS. Furthermore, LNR may eliminate the variation in dissection techniques by different surgeons and the variation in specimen examination by different pathologists and is more accurate than lymph node status for prognosis prediction.

Apart from LNR, the proposed nomograms contain several other predictors, such as age, sex, grade, chemotherapy and the number of lymph nodes removed. Our research identified that older age was strongly associated with worse survival, as shown in other reports (3, 4, 28). Similar to the conclusion of Wang et al. (29), the mortality rate of women was lower than that of men. Moreover, histologic grade, which reflects the biological characteristics of tumour tissue, was also an important prognostic factor for PDA patients in our study. This was consistent with previous reports (3, 28, 30, 31). Ecker et al. (32) indicated that PDA patients with lymph node positivity after surgery could gain survival benefits from adjuvant chemotherapy. A similar effect was observed in our research. In addition, this study demonstrated that the number of lymph nodes examined ≥ 15 was associated with improved survival, which was also in accordance with the results of some investigations (20, 33, 34). The possible reason is that the number of lymph nodes removed affects not only the accuracy of N staging but also the therapeutic effect of lymphadenectomy to eradicate potentially hidden lesions in lymph nodes (20).

In the present study, we constructed and validated nomograms to predict 1-, 3-, and 5-year OS and CSS for PDA patients after surgery. The nomograms have the following advantages. First, their prediction ability was superior to that of the AJCC TNM staging system (8th edition). This is because our nomograms integrated not only clinicopathological variables but also demographic variables, which were more accurate than the TNM staging system. DCA curves confirmed this. Second, the variables used in the nomograms are easily obtained in clinical work. Therefore, by using the variable scores, clinicians may precisely assess the risk factors for patients and develop subsequent individualized treatment. A clear risk stratification of patients in different stages was demonstrated by survival curves. Third, the SEER database is a large and open database. Its information comes from 18 states in the US. Thus, the extracted data were rich, detailed and multicentric. Our results should be more applicable to the general population than studies from a single centre.

However, this study has several limitations. First, our nomograms were constructed based on the SEER database. SEER only includes information from American patients, which may not represent patients from all over the world. Second, some important parameters, such as surgical margin status, vascular invasion, chemotherapy regimens and radiation dose/technology, were unavailable in the SEER dataset. This may have an impact on some results.

In conclusion, this study demonstrated that the LNR is an independent risk indicator for PDAs. Based on the SEER database, we constructed nomograms to predict OS and CSS for PDAs after surgery. These nomograms could assist clinicians in making individualized predictions of patient survival and providing improved treatment suggestions.

## Data availability statement

The original contributions presented in the study are included in the article/**Supplementary Material**. Further inquiries can be directed to the corresponding authors.

## Author contributions

All authors helped to perform the research; JS: performed the literature search and wrote the manuscript. SL and JC: performed data collection. SL and SS: contributed to statistical analysis. JZ and YW: contributed to revising the manuscript and study supervision. All authors contributed to the article and approved the submitted version.

## Funding

This work was supported by the Tianjin Health Bureau under Grant (No. 2014KR04) and the Key Research Project of Tianjin Health and Family Planning Commission (No. 15KG114).

## Acknowledgments

The authors sincerely thank the Surveillance, Epidemiology, and End Results (SEER) program for providing high-quality open resources for researchers.

## Conflict of interest

The authors declare that the research was conducted in the absence of any commercial or financial relationships that could be construed as a potential conflict of interest.

## Publisher’s note

All claims expressed in this article are solely those of the authors and do not necessarily represent those of their affiliated organizations, or those of the publisher, the editors and the reviewers. Any product that may be evaluated in this article, or claim that may be made by its manufacturer, is not guaranteed or endorsed by the publisher.
