# Supplementary figures and images for: Development and validation of lymph node ratio-based nomograms for primary duodenal adenocarcinoma after surgery
Source: Front Oncol. 2022 Oct 4;12:962381. doi: 10.3389/fonc.2022.962381 (PMC9584089; doi:10.3389/fonc.2022.962381)

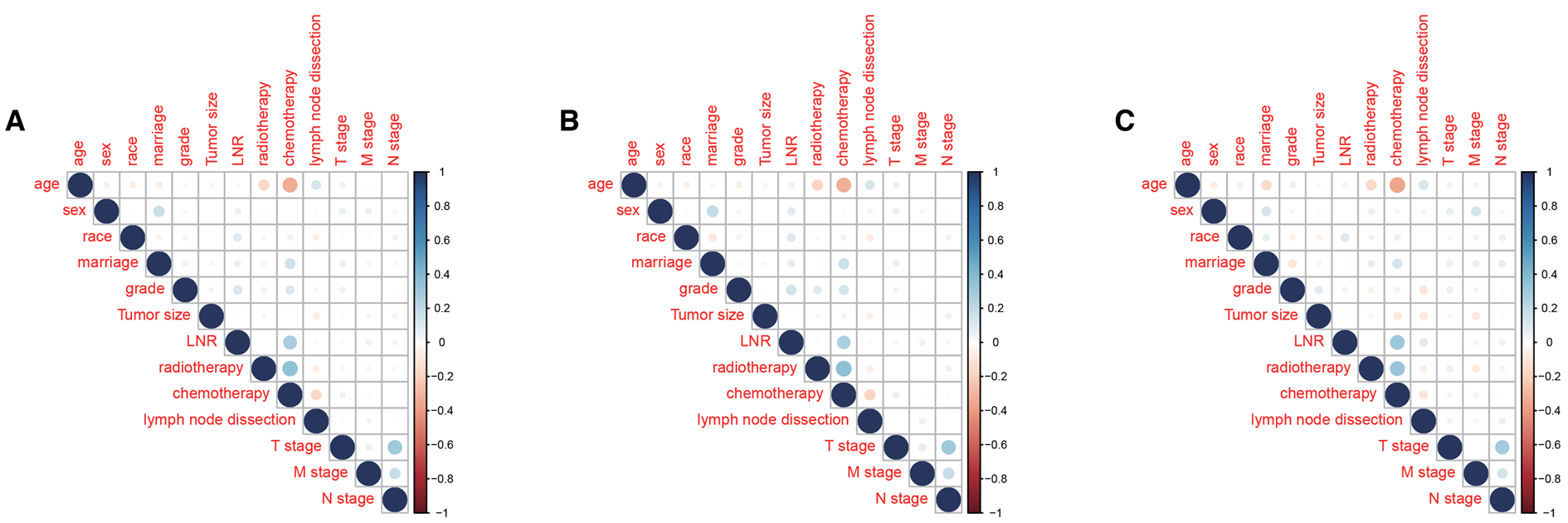

Supplement: Supplementary file 1 [file Image_1.tif]

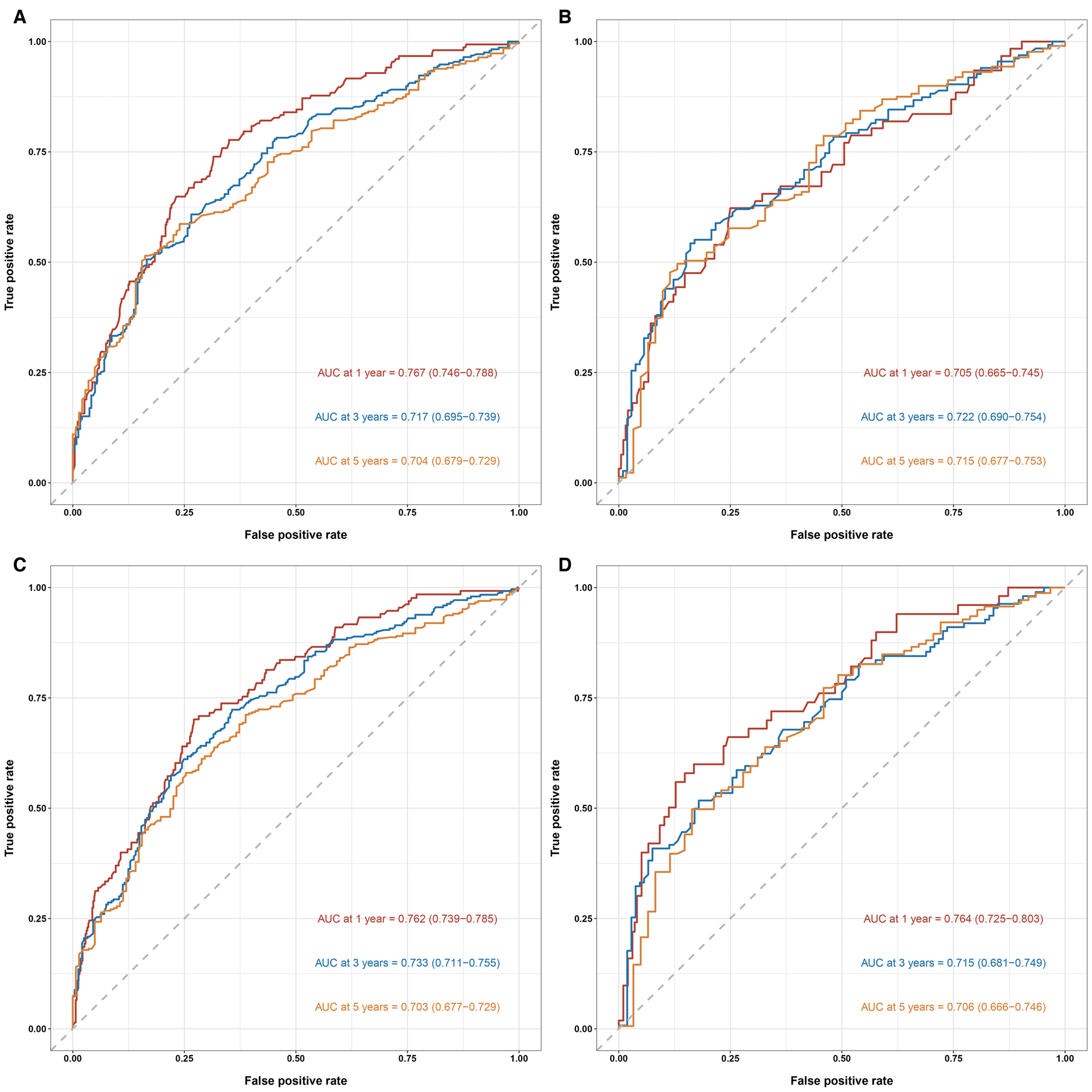

Supplement: Supplementary file 2 [file Image_2.tif]
