# Supplementary material for: Development and validation of lymph node ratio-based nomograms for primary duodenal adenocarcinoma after surgery
Source: Front Oncol. 2022 Oct 4;12:962381. doi: 10.3389/fonc.2022.962381 (PMC9584089; doi:10.3389/fonc.2022.962381)
Supplement: Supplementary file 3 [file Table_1.doc]

**SUPPLEMENTARY MATERIAL**

**Figure S1.** Correlation analysis between different variables in the overall dataset (A), the training set (B) and the validation set (C).

**Figure S2.** Receiver operating characteristic curves (ROCs) of the nomogram for OS prediction (A, training set; B, validation set) and CSS prediction (C, training set; D, validation set). OS, overall survival; CSS, cancer-specific survival; AUC, area under the receiver operating characteristic curve. For ROCs, red represents 1 year, blue represents 3 years and yellow represents 5 years.
